# Substrate *O*‐glycosylation actively regulates extracellular proteolysis

**DOI:** 10.1002/pro.5128

**Published:** 2024-07-29

**Authors:** Elizabeta Madzharova, Fabio Sabino, Konstantinos Kalogeropoulos, Chiara Francavilla, Ulrich auf dem Keller

**Affiliations:** ^1^ Department of Biotechnology and Biomedicine Technical University of Denmark Kongens Lyngby Denmark

**Keywords:** crosstalk, degradomics, glycosylation, LC–MS, MMP9, post‐translational modifications, proteolysis, proteomics, TAILS, Tn antigen

## Abstract

Extracellular proteolysis critically regulates cellular and tissue responses and is often dysregulated in human diseases. The crosstalk between proteolytic processing and other major post‐translational modifications (PTMs) is emerging as an important regulatory mechanism to modulate protease activity and maintain cellular and tissue homeostasis. Here, we focus on matrix metalloproteinase (MMP)‐mediated cleavages and *N*‐acetylgalactosamine (GalNAc)‐type of *O*‐glycosylation, two major PTMs of proteins in the extracellular space. We investigated the influence of truncated *O*‐glycan trees, also referred to as Tn antigen, following the inactivation of C1GALT1‐specific chaperone 1 (COSMC) on the general and MMP9‐specific proteolytic processing in MDA‐MB‐231 breast cancer cells. Quantitative assessment of the proteome and N‐terminome using terminal amine isotopic labelling of substrates (TAILS) technology revealed enhanced proteolysis by MMP9 within the extracellular proteomes of MDA‐MB‐231 cells expressing Tn antigen. In addition, we detected substantial modifications in the proteome and discovered novel ectodomain shedding events regulated by the truncation of *O*‐glycans. These results highlight the critical role of mature *O*‐glycosylation in fine‐tuning proteolytic processing and proteome homeostasis by modulating protein susceptibility to proteolytic degradation. These data suggest a complex interplay between proteolysis and *O*‐GalNAc glycosylation, possibly affecting cancer phenotypes.

## 1 INTRODUCTION

The extracellular matrix (ECM) represents a dynamic cellular microenvironment, increasingly recognized not solely as a scaffold for structural support but as a crucial modulator of different cellular processes (Valdoz et al., 2021). Constituted by a functional network of secreted and membrane‐bound proteins, glycoproteins, and proteoglycans, the ECM surpasses its conventional function by mediating cellular behaviours, including proliferation, differentiation, migration, adhesion, and signal transduction, emphasizing the ECM's contribution to tissue homeostasis and physiological regulation (Dzobo & Dandara, 2023; Karamanos et al., 2021; Yuan et al., 2023).

ECM remodelling and functional regulation are substantially mediated through proteolysis, governed by a proteolytic network encompassing disintegrin and metalloproteases (ADAMs), matrix metalloproteinases (MMPs), and different serine and cysteine proteases. These proteases exert their functions by irreversibly processing different biomolecules (growth factors, cell surface receptors, signalling molecules, etc.), which initiates a sequence of molecular cascades essential for preserving a dynamically regulated extracellular environment (Kollet et al., 2024; Vidak et al., 2019). Therefore, precise regulation of the activity of the proteases is critical, given that dysregulation of proteolytic activities can underlie different pathological conditions, including inflammatory disorders and cancer (Afshar et al., 2023; Park et al., 2020).

Several mechanisms for regulating protease activity have been characterized, including regulation of protease gene expression, expression of protease precursors (zymogens), distinct cellular compartmentalization of proteases and their substrates, regulation through endogenous inhibitors, and the modification of proteases and substrates through post‐translational modifications (PTMs) (Clark et al., 2008; Goettig, 2016; Rose et al., 2021; Yamamoto et al., 2015). Within the spectrum of mechanisms that regulate proteolytic processing, the contribution of PTMs is particularly significant, establishing an additional dimension of nuanced regulation of protease activity. PTMs can intricately modulate protease functionality, facilitating either the direct activation or inactivation of proteases or altering substrate specificity (Goettig, 2016). Moreover, it has been increasingly acknowledged that PTMs, when present on substrates, have a substantial capacity for differential regulation of protease activity. In recent developments, among the different PTMs, *O*‐GalNAc glycosylation of substrates has emerged as a prevalent modulator of proteolysis (Gill et al., 2011; Goth et al., 2018; King et al., 2018; Schjoldager & Clausen, 2012). *O*‐GalNAc glycosylation is a unique type of protein glycosylation, identified mainly on extracellular and secreted glycoproteins (Spiro, 2002; Tian & Ten Hagen, 2009). Functional modification by substrate *O*‐GalNAc glycosylation was demonstrated for proteases from different classes, suggesting that combinatorial crosstalk can occur independently of the mechanism of catalysis (Birch et al., 1991; Goth et al., 2018; King et al., 2018; Madzharova, Kastl, et al., 2019). For example, the interaction between *O*‐glycosylation and proteolysis regulates the function of fibroblast growth factor 23 (FGF23), a key regulator of phosphate homeostasis (Ramon et al., 2010). Glycosylation at the proprotein convertases (PCs) processing site interferes with the protease activity and hampers FGF23 processing (Kato et al., 2006; Schjoldager & Clausen, 2012). Similarly, *O*‐glycosylation at the PC's cleavage site interferes with the transformation of proopiomelanocortin into its active form by PC enzymes (Birch et al., 1991). Furthermore, others have shown that *O*‐glycosylation critically influences ADAMs‐mediated ectodomain shedding of various substrates, including tumour necrosis factor‐alpha (TNF‐α), interleukin‐6 receptor (Il‐6R), heparin‐binding epidermal growth factor‐like growth factor (HB‐EGF), suggesting a potential role of *O*‐GalNAc in fine‐tuning cellular processes such as cell survival, proliferation, differentiation, and apoptosis (Goth et al., 2015; Minond et al., 2012). Additionally, the modification of the G‐protein‐coupled receptor (GPCR), the β1‐adrenergic receptor, through site‐specific *O*‐glycosylation, showcases a broader regulatory function of glycosylation, which co‐regulates ADAM17 proteolytic processing of this receptor, thereby altering the receptor's processing, its functionality, and its impact on cellular behaviour (Goth et al., 2017; Hakalahti et al., 2010; Lackman et al., 2018).

Given the emerging evidence that site‐specific *O*‐glycosylation can modulate proteolysis, we investigated how the MMPs, essential proteases in ECM remodelling, are affected by the *O*‐GalNAc glycosylation of their substrates (Madzharova, Kastl, et al., 2019). We specifically focused on MMP9, which is strongly associated with carcinogenesis. For example, MMP9's role as a potential prognostic marker and therapeutic target has been studied and suggested in breast cancer (Huang, 2018; Kalavska et al., 2020; Kwon, 2023; Rashid & Bardaweel, 2023). Therefore, assessing the regulatory aspect of the interplay between substrate glycosylation and MMP9 proteolytic processing will aid in dissecting additional layers of regulation of this cancer‐related protease (Huang, 2018; Rodríguez et al., 2010; Vandooren et al., 2013).

This study assessed the modulation of MMP9 proteolytic processing by substrate *O*‐GalNAc glycosylation in human breast adenocarcinoma cells. Initially, we exploited the power of advanced cellular engineering to knockout the COSMC gene, a molecular chaperone essential for active T‐synthase, the enzyme that controls the *O*‐glycan elongation (Ju & Cummings, 2002). In various cancers, the loss of COSMC function leads to the expression of short glycans referred to as Tn antigens, characterized by a simple α‐GalNAc linked to Serine/Threonine (Ser/Thr), which promotes oncogenic properties (Radhakrishnan et al., 2014; Rømer et al., 2021). This approach, known as the SimpleCell strategy, has enabled us to access substrate proteomes expressing truncated *O*‐glycans (Yang et al., 2014). We monitored the MMP9‐dependent proteolytic processing in secreted proteomes, expressing either normal or truncated *O*‐glycans. In addition, we evaluated the impact of *O*‐glycosylation on the overall proteome and investigated whether alterations in substrate *O*‐glycosylation may generally affect proteolysis. Our results demonstrate that *O*‐GalNAc glycosylation significantly modulates proteolysis. Specifically, truncated glycans were associated with enhanced proteolysis and a greater susceptibility to proteolytic cleavage. Overall, this dataset offers profound insights into the complex mechanisms of ECM regulation and the role of *O*‐glycosylation in these processes.

## 2 RESULTS

### 2.1 *O*‐glycosylation modulates MMP9 processing

**FIGURE 1:**
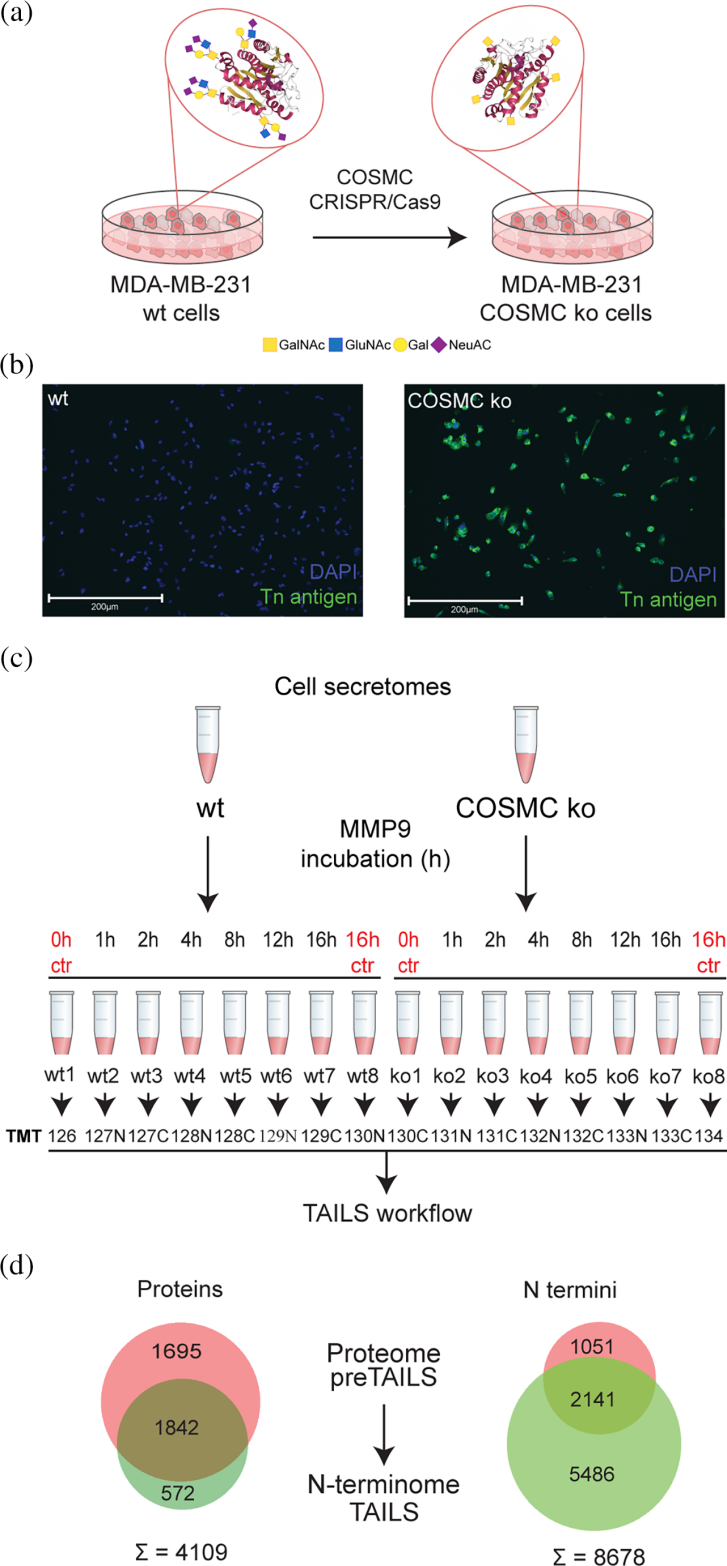
Proteome and N‐terminome analysis of wt and COSMC ko MDA‐MB‐231 cells. (a) COSMC was genetically inactivated by CRISPR/Cas9 to generate MDA‐MB‐231 COSMC ko with truncated *O*‐glycan trees. GalNAc, *N*‐acetyl‐d‐galactosamine; GlcNAc, *N*‐acetyl‐d‐glucosamine; Gal, d‐galactose; NeuAc, *N*‐acetylneuraminic acid. (b) Immunofluorescence analysis of Tn antigen confirmed functional inactivation in COSMC KO cells. DAPI was used to visualize cell nuclei. Scale bar: 200 μm. *N* = 2. (c) Cell secretomes were collected from wt and COSMC ko MDA‐MB‐231 cells, incubated with active recombinant human MMP9 for increasing time points, and analyzed by 16plex‐TMT‐TAILS. Individual samples from the time‐series experiments were treated as replicates in quantitative comparisons between genotypes. (d) Venn diagram illustrating the distribution and overlap of the identified 4109 proteins and 8678 N termini between the preTAILS and TAILS samples.

To study the crosstalk between *O*‐glycosylation and limited proteolysis, we generated MDA‐MB‐231 breast cancer cells with modified *O*‐glycan structures (Figure 1a). For this, we followed the SimpleCell strategy and engineered cells deficient in COSMC using CRISPR/Cas9 genome editing (Steentoft et al., 2013). The expected genetic modification of the COSMC gene was verified by Sanger genome sequencing (Figure S1). We showed functional inactivation of T‐synthase by immunofluorescence staining for Tn antigen, which was only detectable upon ablation of COSMC in MDA‐MB‐231 knock‐out (ko) but absent from wild‐type controls (wt) (Yang et al., 2014) (Figure 1b, Figure S1). Next, we collected secretomes from wt and COSMC ko MDA‐MB‐231 cells, incubated them with activated human recombinant MMP9 for 1, 2, 4, 8, 12, and 16 h in comparison to controls at 0 and 16 h, and subjected all samples to 16plex‐TMT‐terminal amine isotopic labelling of substrates (TAILS) mass spectrometry analysis (Figure 1c). We identified 3537 proteins (Table S1) and 3192 unique N termini (Table S2) from the preTAILS and 2414 proteins (Table S3) and 7627 unique N termini (Table S4) from the TAILS sample, respectively, summing up to a total of 4109 identified proteins and 8678 N‐terminal peptides (Figure 1d).

**FIGURE 2:**
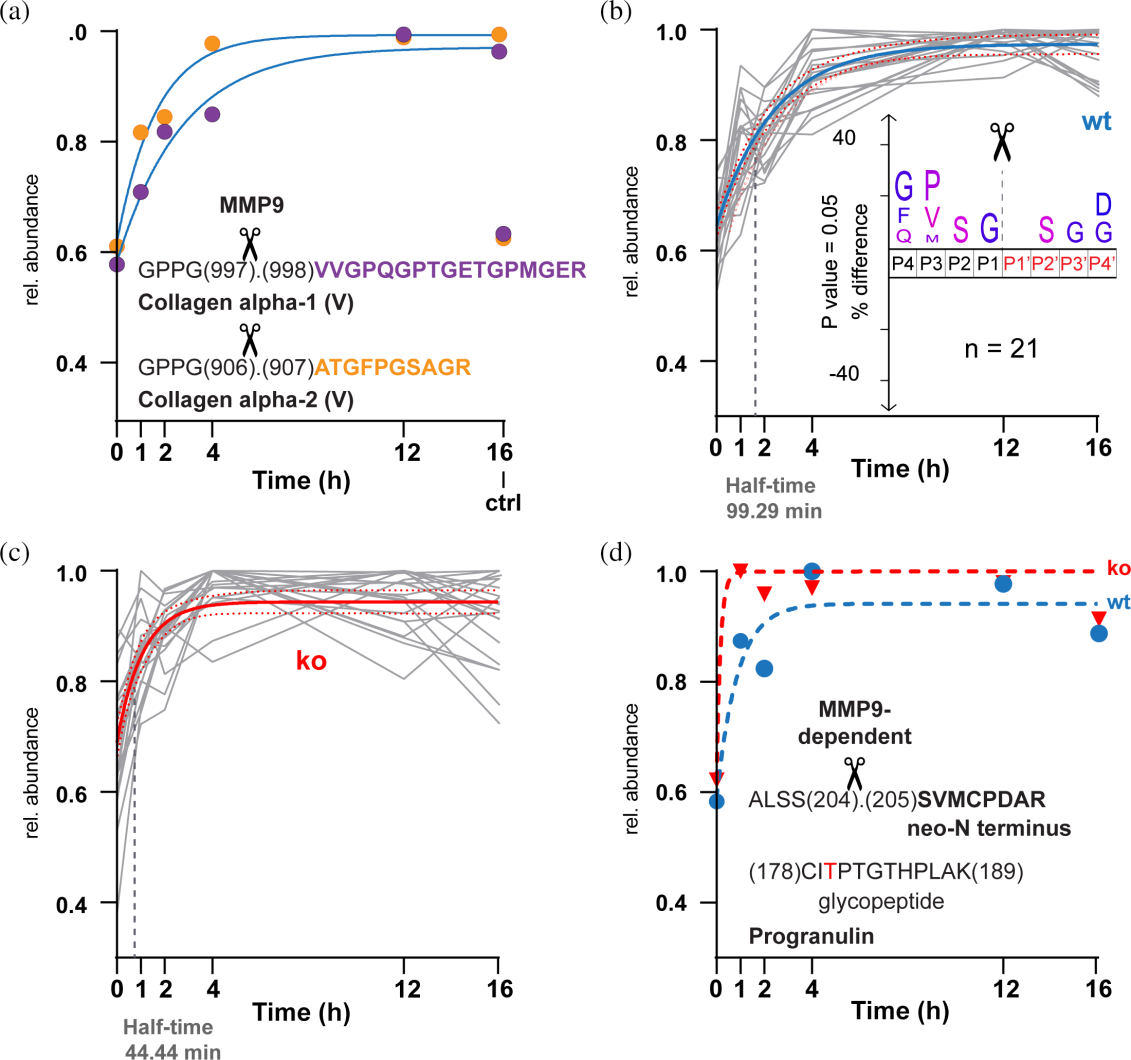
MMP9‐time dependent processing in wt and ko cell secretomes. (a]) Known MMP9 cleavages have been extracted from the wt neo‐N termini dataset after normalization of abundance values to the maximum of wt time‐series. Values for respective neo‐N‐terminal peptides are color‐coded (purple: VVGPQGPTGETGPMGER, orange: ATGFPGSAGR), and curves have been fit following pseudo‐first‐order kinetics including all values but the 16 h control. (b,c) Abundance profiles in (a) have been used as templates to extract similar profiles (Euclidean distance; 10 closest feature/template feature) from the wt time (b) or ko (c) series. The shared curve has been fitted with best‐fit values for all datasets in the graph. Values for 16 h control are not shown. Half‐time indicates the half‐time parameter of the fitted curve. Inset in (b) shows IceLogo analysis of the 21 cleavage sites corresponding to neo‐N termini represented by the abundance profiles. (d) Data for MMP9‐dependent processing of progranulin has been extracted from graphs shown in (b) and (c) (blue: Wt, red: Ko) and individually fitted by pseudo‐first‐order kinetics. The shift indicates increased processing rate of mutant with truncated *O*‐glycan tree (ko) close to a known glycosite (in red).

Next, we looked for MMP9‐dependent cleavage events in our time‐resolved TAILS dataset. To compare time‐dependent changes in abundances of neo‐N termini in samples from both the wt and ko genotypes, we separately normalized relative amounts of neo‐N‐terminal peptides to the maximum of each time series, either recorded using samples from wt or ko cells but within the same multiplexed experiment (Table S5). Next, to validate MMP‐9‐dependent processing, we mined the dataset for known and typical MMP9 cleavages and identified two MMP9 cleavage sites in the collagen V alpha‐1 and alpha‐2 chain, which are known substrates of MMP9 (Kridel et al., 2001; Niyibizi et al., 1994) (Figure 2a). Both these cleavage sites were characterized by a time‐dependent increase in the amount of the resulting neo‐N‐terminal peptide in samples from the wt cells using pseudo‐first‐order kinetics for both cleavage sites (Schlage et al., 2014) (Figure 2a, with collagen V alpha‐1 in purple and collagen V alpha‐2 in orange). To identify MMP9‐dependent cleavage events from the wt and ko time series, we used the abundance profiles of collagen V alpha‐1 and alpha‐2 chain cleavages as templates. We extracted 21 distinct cleavage events across 21 proteins in the wt time series (Figure 2b). The enrichment of proline in P3, which is indicative of MMP9's cleavage specificity, suggests that these cleavages could be related to an MMP9 specificity profile (Eckhard et al., 2016a, 2016b). However, among these cleavages, many events did not match the MMP9 specificity profile, suggesting that MMP9 orchestrates additional proteolytic activity by activating or modulating the activity of other proteases. When we compare the 21 abundance profiles from the wt time series with time‐resolved abundance data for the same neo‐N termini obtained from samples of COSMC ko cells, we recognize a general trend toward increased processing rates. This is indicated by a reduced half‐time parameter (from 99.29 min in wt to 44.44 min in ko cells) for a pseudo‐first‐order kinetics curve with best‐fit values for all 21 profiles (Figure 2c). Two of these neo‐N termini were identified in known glycoproteins (EGF‐containing fibulin‐like ECM protein 1 and progranulin) (Table S5) with cleavage sites in proximity (less than 30 residues) to known glycosites, identified by Steentoft et al. (2013). As exemplified for progranulin in Figure 2d, MMP9‐dependent limited proteolysis of progranulin at Ser^204^ (located near a previously identified glycopeptide, CITPTGTHPLAK) was moderate, but increased in samples from ko cells, which have truncated *O*‐glycan trees, compared to the wt cells. Together, these data demonstrate the role of *O*‐glycosylation in modulating MMP9's proteolytic activities, both directly and indirectly.

### 2.2 COSMC knockout significantly changes the *O*‐glycosylation machinery and protease network in MDA‐MB‐231

**FIGURE 3:**
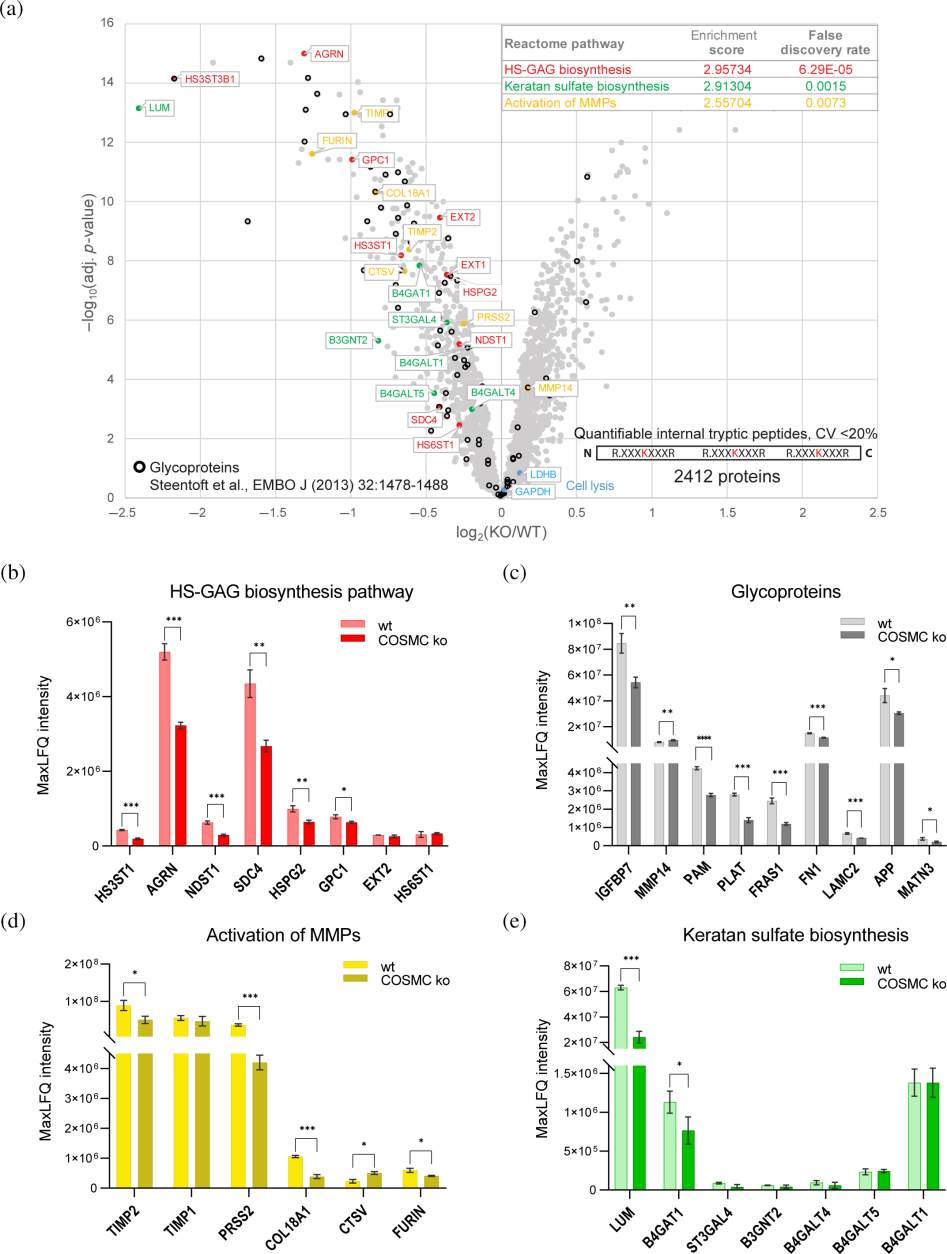
Differential protein abundance in wt and ko cells. (a) Volcano plot showing proteins quantified using only TMT‐labeled internal tryptic peptides with CV <20% between samples from either wt or ko cells (moderated *t*‐statistics [limma], adj. *p*‐value [Benjamini‐Hochberg]). Pathway assignment is color‐coded based on the analysis shown in the inset. GAPDH and LDHB, annotated as markers for cell lysis, are shown in blue. Proteins identified as glycoproteins in MDA‐MB‐231 secretomes by Steentoft et al. are marked by black circles (Steentoft et al., 2013). The inset shows the functional enrichment analysis of the quantified proteins with decreased abundance in COSMC ko, using STRING v11 and Reactome pathways. B‐E Relative abundance of pathway‐associated proteins (b,d,e) and glycoproteins (c) identified in the proteome analyzed with the DIA method. Bars indicate means ± SD. The data presented in each graph is derived from *N* = 3 independent technical replicates. Two‐tailed *t*‐test, *p < 0.05; **p < 0.01; ***p < 0.001.

Since *O*‐glycosylation affects a myriad of proteins and impacting GalNAc‐type‐*O*‐glycan Core 1 by genetic ablation of COSMC function has been shown to alter cancer cell phenotypes significantly, we next compared global protein abundances in wt and ko cells (Hofmann et al., 2015; Schjoldager et al., 2020). We focused on 2412 proteins identified and quantified with high confidence (e.g., by only using lysine‐containing peptides and thus quantifiable internal tryptic peptides) in the preTAILS dataset (Table S6). As we excluded any proteins quantified by quantifiable internal tryptic peptides with a coefficient of variance >20% between samples from individual time points, we treated each time point of the MMP9 time‐series experiment as a separate replicate (Figure 1b; wt1‐8, ko1‐8, with the exclusion of wt5 and ko5, following a quality inspection). Using moderated *t*‐test statistics (limma) and Benjamini‐Hochberg (BH) multiple testing correction, we identified significantly differential abundances of 137 proteins (fold‐change ±1.5, *p*‐value <0.05), of which 38 were higher and 99 lower in abundance in samples from ko than wt cells, respectively (Figure 3a table, Table S6). Interestingly, functional enrichment analysis using STRING v11 revealed the highest enrichment scores for pathways associated with carbohydrate and glycan metabolism, including galactosyltransferases, that were all significantly lower in abundance upon functional inactivation of COSMC (Figure 3a inset, Table S7), which suggests unrecognized functions of truncated *O*‐glycans in regulating of cellular glycan homeostasis and proteolytic networks (Szklarczyk et al., 2019). Moreover, proteins associated with the MMP activation pathway were also decreased in abundance, suggesting a role for *O*‐glycosylation in proteolytic systems. Finally, known glycoproteins that had been identified in secretomes from MDA‐MB‐231 cells by Steentoft et al. and 88 of which could be overlaid with our dataset were almost exclusively lower in abundance in samples from ko than from wt cells (Steentoft et al., 2013) (Figure 3a). We selected their study as a reference due to their use of the same cell line and SimpleCell strategy for glycoprofiling, thereby ensuring consistency in our experimental approach. Among them, we identified fibronectin, insulin‐like growth factor‐binding protein 7 (IGFBP7) and tissue‐type plasminogen activator (PLAT), which are all glycoproteins intricately associated with cancer development and progression (Corte et al., 2005; Gibson et al., 2023; Godina et al., 2021; Haley & Freeman., 2018; Jin et al., 2020). These data suggest that elongated *O*‐glycan trees may affect protein stability and/or expression as well as the mechanisms by which these proteins influence carcinogenesis. Notably, the observed differences were not due to differential cell lysis efficiency between genotypes. Indeed, the abundance of glyceraldehyde 3‐phosphate dehydrogenase (GAPDH) and lactate dehydrogenase (LDHB), which are widely used as markers for cell lysis, were similar in wt and ko cells (Chan et al., 2013) (Figure 3a, Table S6). To validate these findings, we conducted a comprehensive analysis of the proteome using quantitative proteomics (Figure 3b–e). The proteins belonging to enriched pathways including heparan sulfate glycosaminoglycans (HS‐GAG) biosynthesis, keratan sulfate biosynthesis and activation of MMPs, as well as the glycoproteins identified as differentially expressed by wt and ko cells (Figure 3a table, Table S6) were also identified and quantified in the proteome using the quantitative data‐independent analysis (DIA) (Figure 3b–e, Table S8, Figures S2 and S3). Specifically, we observed a decreased abundance of proteins belonging to the carbohydrate metabolism pathways and glycoproteins in ko compared to wt cells (Figure 3b,c) and alterations in the MMPs activation pathway (Figure 3d, Table S8). However, DIA analysis did not validate the decreased abundance of proteins belonging to the keratan sulfate biosynthesis pathway except lumican (LUM) and beta‐1,4‐glucuronyltransferase 1 (B4GAT1) (Figure 3e, Table S8). Overall, our data indicate the impact of truncated *O*‐glycans on the *O*‐glycosylation machinery and protease activity/expression (Figure 3).

### 2.3 Lack of extended *O*‐glycan trees result in increased limited proteolysis

**FIGURE 4:**
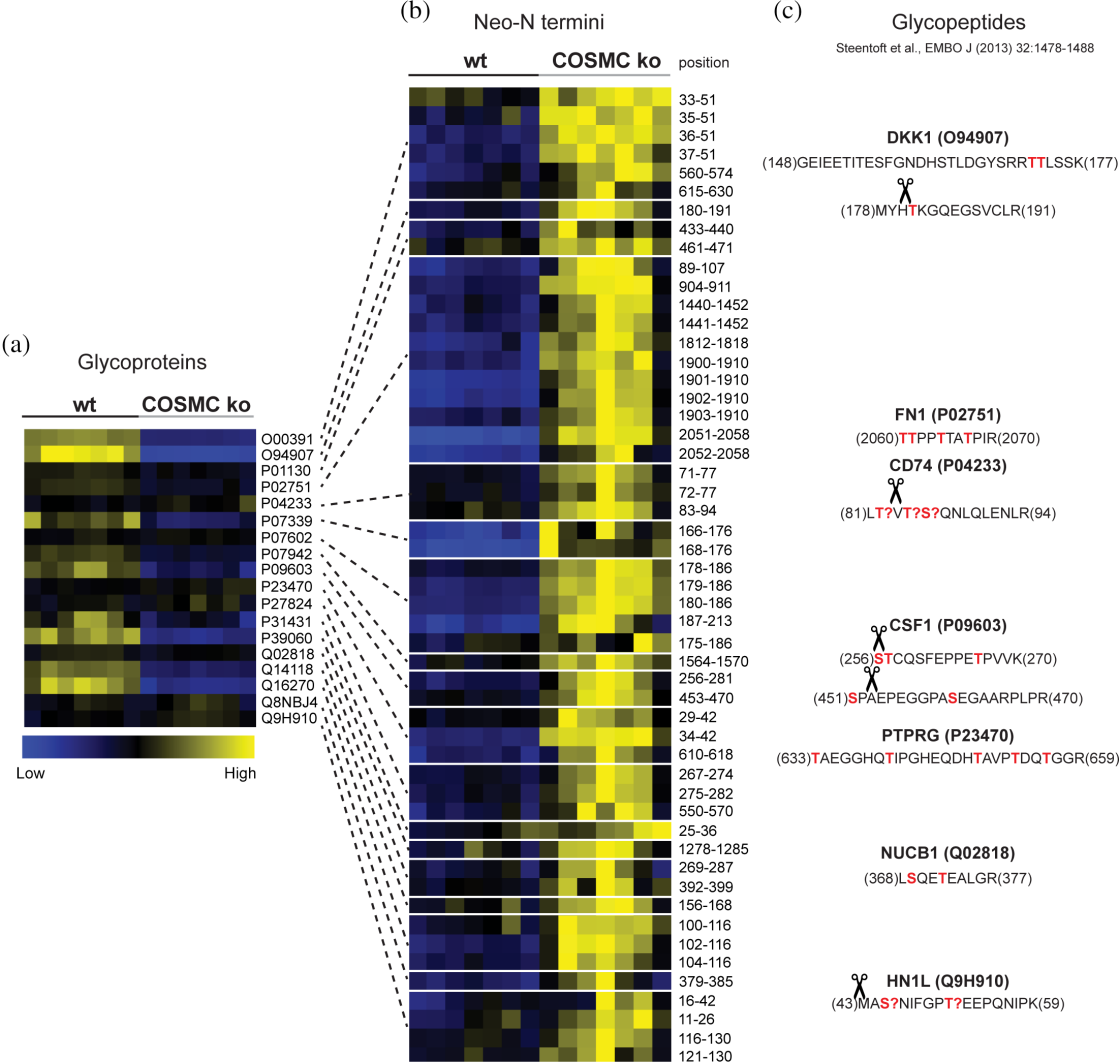
Differential proteolytic processing of glycoproteins in samples from wt and COSMC ko cells. (a) Glycoproteins, as marked in Figure 3a with a log2 (ko/wt) abundance ratio <0.2, were extracted and correlated (as indicated by lines) with (b) their respective identified neo‐N termini, which showed at least a 20% increase in abundance in samples from ko compared to wt cells. Time‐series samples were treated as replicates for genotype quantitative comparisons. “Position” indicates the position of the neo‐N terminus based on the UniProt fasta sequences. (c) Correlation of identified neo‐N termini with glycopeptides extracted from Steentoft et al. highlighted cleavages near annotated glycosites (in red, “?” denotes for unassigned sites) in DKK1 (O94907), FN1 (P02751), CSF (P09603), CD74 (P04233), and PTPRG (P23470) proteins.

Having assessed the effects of *O*‐glycan truncation on glycoprotein abundances in MDA‐MB‐231 breast cancer cells, we wondered if changes to protein *O*‐glycosylation generally alter proteolytic processing, potentially leading to enhanced proteolysis. To do so, we focused on known glycoproteins in our dataset tables (Table S6) and correlated their relative abundances (Figure 4a) with levels of related neo‐N termini (Figure 4b, Tables S2 and S3) in samples from both genotypes. Since the vast majority of glycoproteins were lower in abundance in samples from ko than from wt cells (Figure 3a, Figure 3c), we focused on 18 (out of 88) glycoproteins with log_2_(ko/wt) abundance ratios of <0.2 and related them to neo‐N termini with significantly (*t*‐test, adj. *p*‐value <0.05 [BH]), and at least 20% higher abundance in ko than wt samples (Figure 4a,b). Reciprocal changes in abundances at protein and neo‐N termini levels indicated bona fide proteolytic processing events. Eight identified cleavages in seven glycoproteins were located close to known glycosylation sites (Figure 4c), suggesting an inhibitory effect of extended *O*‐glycan trees on limited proteolysis. Proteins subjected to cleavage near identified glycosylation sites included secreted glycoproteins (DKK1, FN1, CSF) as well as membrane‐anchored proteins (CD74, PTPRG), whereby for the latter, glyco‐dependent cleavages were close to ectodomain shedding sites. Together, these results provided evidence for fine‐tuning limited proteolysis by protein *O*‐glycosylation with an influence on functional modification, such as releasing bioactive ectodomain from the cell surface.

### 2.4 Cathepsin S‐mediated glyco‐dependent proteolytic processing of CD74

**FIGURE 5:**
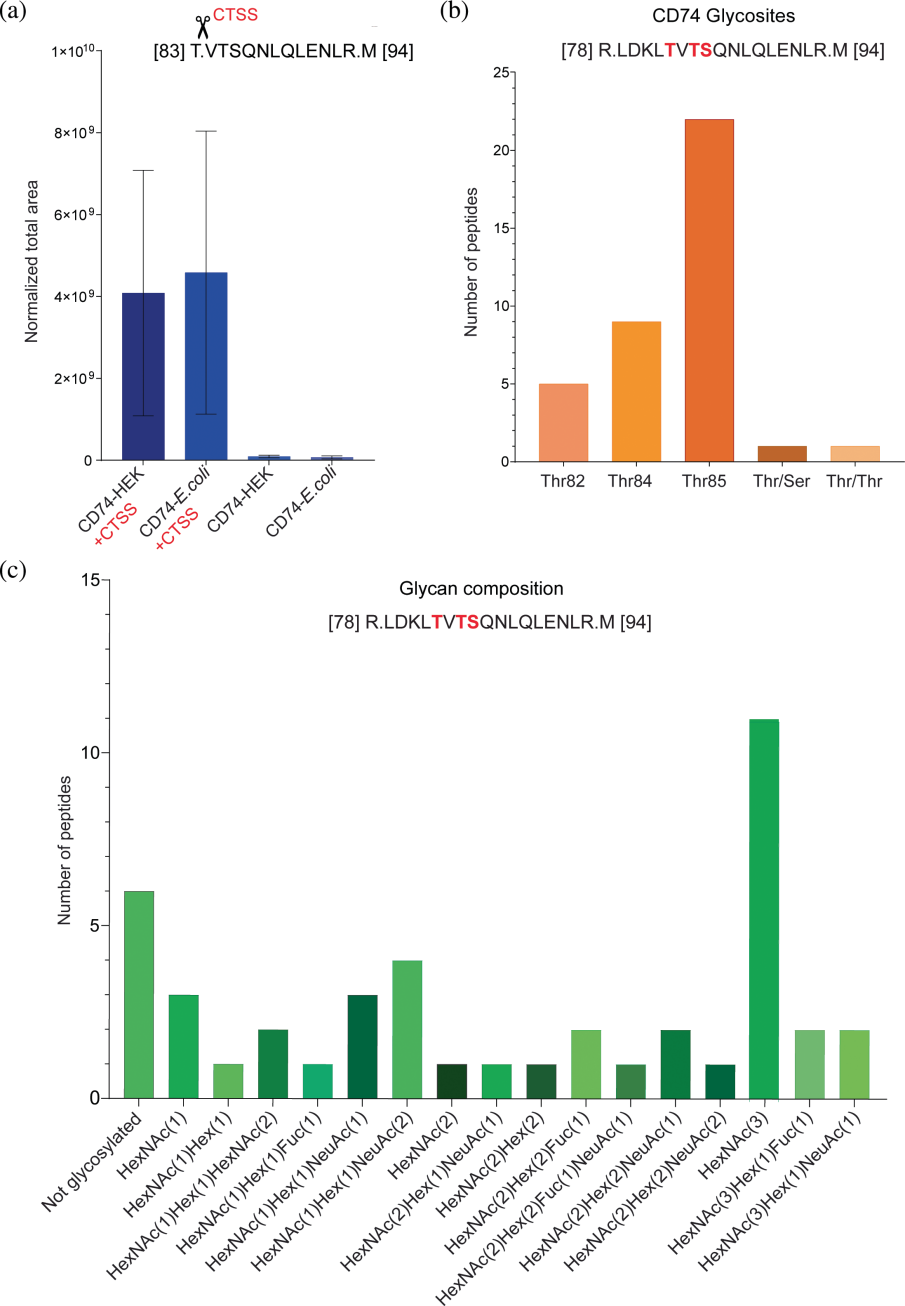
Validation of CTSS‐mediated cleavage of CD74 via PRM. (a) Recombinant human CD74 proteins in both glycosylated (produced in HEK cells) and non‐glycosylated (produced in *E. coli*) forms were treated with activated recombinant human CTSS. Control samples for each protein type without CTSS were included. The presence of the semi‐tryptic peptide VTSQNLQLENLR was quantified using PRM. The bar graph shows the total ion current (TIC) normalized area‐under‐the‐curve (AUC) for this specific peptide across the samples (*n* = 2), presented as mean ± SD. (b) Glycoproteomics profiling of the CTSS cleavage site‐containing LDKLTVTSQNLQLENLR peptide on the recombinant human protein CD74. The bar graph displays glycosylation at three distinct sites Ser85, Thr82, and Thr84 (highlighted in red)—on the peptide, showing the specific locations and the frequency of glycan attachments. (c) Glycoproteomics profiling of the CTSS cleavage site‐containing LDKLTVTSQNLQLENLR peptide reveals the variety of glycan compositions found within the peptide's sequence. Hex, hexose; HexNAc, *N*‐acetylhexosamine; Fuc, fucose; NeuAc, *N*‐acetylneuraminic acid.

To validate the glyco‐dependent cleavages highlighted in Figure 4c, we focused on the cleavage event observed in membrane‐anchored CD74. This cleavage coincides with the previously identified ectodomain shedding site of Cathepsin S (CTSS) in lysosomal CD74, which is related to its function in antigen processing (Dheilly et al., 2020). Additionally, prior studies indicate that CD74 undergoes *O*‐glycosylation at three distinct positions, Serine(Ser) (Vasudevan & Haltiwanger, 2014), Threonine(Thr)^82^, and Thr^84^, adjacent to the identified cleavage site (Steentoft et al., 2013) (Figure 4c). To investigate the effect of *O*‐glycosylation on the ectodomain shedding of CD74, we incubated recombinant glycosylated and non‐glycosylated versions of CD74 with human recombinant CTSS, including two control samples without CTSS, followed by parallel reaction monitoring (PRM) analysis (Figure S4). The semi‐tryptic peptide VTSQNLQLENLR from CD74, resulting from CTSS cleavage, was more abundant in the non‐glycosylated sample (Figure 5a, Table S9), suggesting that the presence of *O*‐glycosylation modulates CD74 proteolytic processing via CTSS. However, the observed difference was not as pronounced as anticipated. We wondered whether the recombinant glycosylated CD74 was glycosylated at the specific position where the cleavage occurs. We analyzed the glycoprofile specific to CD74 by glycoproteomics and focused only on the HEK‐produced CD74 recognized for its glycosylation, distinguishing it from the *Escherichia coli*‐produced protein variant that lacks glycosylation (Table S10). The fully tryptic peptide LDKLTVTSQNLQLENLR, which contains the CTSS cleavage site, revealed specific glycans attached to Ser^85^, Thr^82^, and Thr^84^ residues of CD74, where most of the identified peptides exhibited glycosylation primarily at the Ser^85^ site (Figure 5b, Table S10). This glycoprofile is consistent with previous glycoproteomics analysis of CD74 (Steentoft et al., 2013). Furthermore, our investigation identified 16 distinct glycan compositions, while specific peptides lacked glycosylation (Figure 5c, Table S10), indicating variability in both site‐specific glycosylation and glycan composition. These findings suggest that site‐specific glycosylation and the glycan composition affect the differential proteolytic processing of CD74.

## 3 DISCUSSION

The comprehension of extracellular proteolysis has evolved from merely facilitating ECM remodelling to being acknowledged as critical for regulating cellular behaviour and homeostasis. This shift imposes reexamining ECM protease regulatory networks to emphasize the importance of precise regulatory mechanisms for balancing their proteolytic activities. This study explored the regulatory dynamics between extracellular proteolysis and *O*‐GalNAc glycosylation, two of the most prominent PTMs in shaping the extracellular proteome. Our findings offer new insights into how physiological *O*‐glycosylation impacts cellular proteome dynamics, revealing that glycan truncation significantly alters the proteome of a model of cancer cells and enhances its susceptibility to proteolytic cleavage. Additionally, various glycans differentially affect the proteolysis process, underscoring the complex regulatory roles of specific glycan structures in cellular proteolysis dynamics. This regulatory mechanism has profound implications in cancer biology, as altered proteolysis and truncated glycan structures are hallmarks of tumorigenesis, suggesting a critical role for these PTMs in cancer progression and metastasis (Radhakrishnan et al., 2014; Radisky, 2024; Rømer et al., 2021).

### 3.1 *O*‐glycosylation directly and indirectly modulates MMP9‐mediated processing

Since MMPs are critical modulators of the extracellular environment in cancer and have been shown to often cleave in the vicinity of *O*‐glycosylation sites, we selected MMP9 to test the modulation of its activity by truncated *O*‐glycan trees specifically (Kessenbrock et al., 2010; King et al., 2018). Compared to its close family member, MMP2, MMP9 processivity is rather low, projecting a limited number of cleavage events that could be identified in our assay (Prudova et al., 2016). In addition, with ~60% of the time‐series maximum, abundances in both controls at 0 and 16 h (no MMP9 incubation) were similar, indicating a relatively high level of endogenous MMP9 in MDA‐MB‐231 secretomes (Figure S5), which has probably masked the effects of the exogenously added protease (Li et al., 2017). While these factors affected coverage of the potential MMP9 substrate space, time‐resolved TAILS analysis ensured high specificity for the identified cleavages (Schlage et al., 2014). The general shift toward increased cleavage upon removal of extended *O*‐glycan trees confirms a protective function of *O*‐glycosylation against uncontrolled proteolysis by MMP9 (Russell et al., 2009). This could play an important role in fine‐tuning MMP9 activity in vivo as MMP9 is dysregulated in tumours with reduced COSMC levels, thereby contributing to the protease's unwanted and detrimental pro‐tumorigenic activities (Kessenbrock et al., 2010). The number of identified cleavage sites with non‐typical MMP specificities further hints toward indirect effects with complex modulation of interconnected protease activities by the extent of *O*‐glycosylation of substrate proteins in the extracellular space.

### 3.2 *O*‐glycosylation is important for protease expression including and beyond MMPs

Our quantitative proteome comparison of MDA‐MB‐231 wt and COSMC ko cells has revealed significant changes in the abundance of proteins known to be modified by *O*‐glycosylation. The lower abundances of glycoproteins following *O*‐glycan tree truncation can be explained by a lack of protection and higher susceptibility to proteolytic degradation or changes in the expression levels of these proteins. This would be in agreement with significant changes to the glycoproteome upon genetic ablation of COSMC in HaCaT keratinocytes (Radhakrishnan et al., 2014). However, our data also indicate additional regulatory mechanisms governed by C1GALT1 activity (Kozarsky et al., 1988). Interestingly, we observed strong alterations in abundances of essential proteases (furin, MMP14) and protease inhibitors (TIMP1, TIMP2), corroborating results on perturbation of the protease web by alterations to proteome *O*‐glycosylation (King et al., 2018). These changes in the protease network can be further supported by the alterations in the abundance of various GAGs, including syndecans, agrin, and glypicans, considering the pivotal role proteolysis plays in their functional optimization through the release of bioactive fragments (Filmus et al., 2009; Huang & Park, 2021; Manon‐Jensen et al., 2010; Mead et al., 2022; Rodríguez‐Manzaneque et al., 2009; Theocharis et al., 2016). Our findings indicate that *O*‐glycosylation might not only govern the stability and expression of these molecules but can also modulate the activity of proteases as MMPs, ADAMs, and PCs, involved in their processing, leading to increased proteolytic susceptibility of the GAGs and, consequently altering the cellular outcomes associated with their functions. Together, these global changes in the proteolytic network potentially contribute to the phenotype of cancer cells with high levels of Tn antigen as a result of impaired COSMC function (Ju et al., 2014). Furthermore, a study by Festari et al. demonstrated that truncated *O*‐glycans in triple‐negative breast cancer reveal a gene expression signature associated with the ECM and proteolysis. This genetic support aligns with our findings, highlighting the extensive consequences of *O*‐glycosylation alterations on the proteolytic network and its role in cancer progression (Festari et al., 2024).

### 3.3 *O*‐glycosylation modulates ectodomain shedding and extracellular functions of CTSS

Analyzing proteins and neo‐N termini using the TAILS approach allowed us to identify bona fide protease cleavage events and their modulation by *O*‐glycan truncation. Increased abundances of neo‐N‐terminal peptides derived from glycoproteins with strongly decreased protein amounts in samples from COSMC ko compared to wt cells might result from enhanced protein degradation. However, most cleavages within focused protein domains indicate specific events rather than global and unspecific processing. In particular, ectodomain shedding of CD74 and PTPRG can be classified as limited proteolysis since both proteins did not change in abundance between genotypes, but there is an increase in the abundance of their neo‐N termini, which is related to the identified cleavage sites. An increase in CD74 cleavage after Thr82 is particularly interesting since it coincides with a known cathepsin S cleavage site in lysosomal CD74, which has been related to its function in antigen processing (Brines, 2004; Rückrich et al., 2006). Our findings broaden the understanding of CTSS cleavage mechanisms by demonstrating that such a cleavage event is not confined to the lysosomal environment but extends to the cell surface, broadening the scope of its effect on different cellular signalling processes. On the cell surface, CD74 acts as a receptor for macrophage migration inhibitory factor (MIF), facilitating the activation of critical signalling cascades such as ERK‐1/2 MAP and PI3K/Akt kinases or impeding p53‐dependent apoptotic pathways (Leng et al., 2003; Richard et al., 2014; Richard et al., 2015; Schröder, 2016; Starlets et al., 2006). Subsequently, mediating the shedding of CD74, CTSS generates a soluble form of CD74 that acts as a decoy receptor, inhibiting MIF binding and altering downstream cellular processes like proliferation and survival. This modulation suggests that cathepsin S has a role in regulating cellular responses that could affect conditions like autoimmune liver disease and melanoma, demonstrating its broad functional implications in cellular signalling (Assis et al., 2014; Fukuda et al., 2022). Additionally, the enzyme's influence extends to interactions between membrane CD74 and TIMP‐1 in breast cancer, promoting internalization and Akt signalling activation (Ebert et al., 2023; Høeberg et al., 2023). The consequences of similar interactions involving soluble CD74 require further exploration, highlighting CTSS's role in modulating diverse signalling pathways across different cellular contexts. More importantly, this ectodomain processing by CTSS is modulated by *O*‐glycosylation, emphasizing the sophisticated interplay between glycan composition and proteolytic processing in dictating the multifunctional roles of CD74. Our data also demonstrate that *O*‐glycosylation modulates CD74 processing, with enhanced shedding observed in the non‐glycosylated CD74 variant. Moreover, distinct glycan structures are shown to differentially regulate this process, illustrating the critical role of specific glycosylation patterns in determining the efficiency and outcome of CTSS ectodomain shedding.

### 3.4 Truncated *O*‐glycans impact the extracellular proteolytic dynamics in breast carcinoma

Truncated *O*‐GalNAc glycans are frequently detected across various cancers, often resulting from disrupted glycosylation pathways caused by differential expression or activity of glycosyltransferases (Ju & Cummings, 2002; Rømer et al., 2021). Their expression significantly facilitates cancer progression and invasiveness, closely linked with poorer clinical outcomes, such as reduced survival rates and increased metastatic potential (Festari et al., 2024; Freitas et al., 2019; Gupta et al., 2020; Pinto & Parameswaran, 2023). In this study, we used the SimpleCells strategy to generate a sample proteome with truncated *O*‐glycans by genetically inactivating COSMC, thereby mimicking the downregulated COSMC expression seen in many cancers that promote oncogenic properties (Dong et al., 2018; Hofmann et al., 2015; Ju et al., 2014; Steentoft et al., 2013).

The truncation of *O*‐glycans significantly influences cellular behaviour, especially given the crucial role of *O*‐glycosylation in protein function (Peixoto et al., 2019; Wandall et al., 2021). It is well‐established that *O*‐glycans impact protein functions by aiding in proper protein folding and trafficking, ensuring accurate cellular localization (Spiro, 2002; Tian & Ten Hagen, 2009; Vasudevan & Haltiwanger, 2014). This PTM also regulates cell surface receptor activity, which involves pathways essential for cell growth, differentiation, and survival. Glycans on cell surface proteins facilitate cell–cell and cell–ECM interactions, adhesion, migration, and invasion‐key processes in cancer metastasis (Freitas et al., 2019; Pinto & Parameswaran, 2023). Additionally, *O*‐glycans enhance protein stability and protect against proteolytic degradation, as observed in mucins and other membrane and secreted proteins, effects also corroborated by our study (Peixoto et al., 2019; Wandall et al., 2021). More importantly, we would like to emphasize with this study that *O*‐glycosylation finely modulates proteolytic processing by regulating the proteolytic network itself and functionally modifying proteins, such as generating bioactive fragments that modulate signalling pathways. This is demonstrated in our study by the shedding of CD74 by CTSS and is supported by findings from other studies as well (Goth et al., 2015; Goth et al., 2017; Hakalahti et al., 2010; Lackman et al., 2018; Minond et al., 2012; Radhakrishnan et al., 2014). With this, we aim to present a novel perspective on *O*‐glycosylation regulation of proteolysis as a highly controlled crosstalk within cellular systems. By mediating selective proteolytic interactions, *O*‐glycosylation orchestrates cellular functions and significantly affects cancer progression.

Lastly, our study complements a study by King et al. that analyzed the consequences of complete removal of *O*‐glycosylation on MMP9 proteolytic processing in MDA‐MB‐231 cells (King et al., 2018). In contrast, we tested the effects of *O*‐glycan truncation, as these short *O*‐glycans can serve as targets for therapeutic intervention and biomarkers for monitoring cancer progression and prognosis (King et al., 2018; Rømer et al., 2021). This could also explain the more subtle differences between samples from wt and glycoengineered cells in our assays, which, however, more closely reflect disease conditions such as tumours with high Tn antigen expression, even though both studies have similar observations (Festari et al., 2024; Rømer et al., 2021).

However, the regulatory aspect of *O*‐glycosylation in proteolytic processing remains largely unexplored. The complexity and specificity of *O*‐glycosylation's influence on proteolysis present a rich area for future investigations, promising insights that could significantly advance our understanding of cancer biology and lead to innovative approaches in cancer treatment.

## 4 MATERIALS AND METHODS

### 4.1 Cell culture and COSMC knockouts

MDA‐MB‐231 cells were purchased from ATCC (HTB‐26) and grown in DMEM media (Merck Life Science A/S, Søborg, DK), supplemented with 1% penicillin/streptomycin (Merck Life Science A/S, Søborg, DK) and 10% fetal bovine serum (FBS, Thermo Fisher Scientific, Roskilde, DK) at 37°C in a 5% CO_2_ atmosphere. The cells were checked monthly for mycoplasma via a PCR‐based detection assay (Venor®GeM—Cambio). The cells were transfected with SpCas9 (BB)‐2A‐GFP construct (Addgene ID: 48138), targeted toward the COSMC sequence 5′ GTGTATGGGGTATACCGCCTT 3′ using Lipofectamine 3000 Reagent (Thermo Fisher Scientific, Roskilde, DK), following manufacturer's instructions. The transfected cells were sorted using BD FACSMelody™ Cell Sorter (Biosciences, USA), after which the single clones were verified by Sanger sequencing, which was outsourced to Eurofins Genomics, Ebersberg, DE (Figure S1). The nucleotide sequences obtained from wt and COSMC cells were aligned using the Align Sequences Nucleotide BLAST tool available from the National Center for Biotechnology Information to detect the induced deletion in the targeted region of the COSMC gene (Figure S1). The COSMC knockout was further characterized by fluorescent immunocytochemistry staining using monoclonal anti‐Tn (SBH Science, Massachusetts, USA) to confirm the presence of truncated *O*‐glycans (Yang et al., 2014). Briefly, MDA‐MB‐231 wt and COSMC ko cells (*N* = 2) were grown in 8‐well chamber slides (Thermo Fisher Scientific, Roskilde, DK) to about 70% confluence. Next, the cells were washed twice with phosphate‐buffered saline (PBS, Thermo Fisher Scientific, Roskilde, DK) and fixed with 4% paraformaldehyde (PFA, Merck Life Science A/S, Søborg, DK) for 10 min at room temperature. The cells were gently washed twice with 1xPBS and permeabilized with 0.5% Triton‐X (Merck Life Science A/S, Søborg, DK) for 5 min at room temperature. After washing three times with PBS, the cells were blocked with 2% bovine serum albumin (BSA, Merck Life Science A/S, Søborg, DK), 0.05% Triton‐X in PBS, for 45 min at room temperature. Tn antibody (1:100, w/w) was added to the cells and incubated overnight at 4°C. Next, the cells were gently washed three times with PBS and incubated with secondary anti‐mouse IgG, horseradish peroxidase (HRP) conjugated antibody (1:500, Abcam, Cambridge, UK) mixed with 4′,6‐diamidino‐2‐phenylindole (DAPI, 1:1000, Thermo Fisher Scientific, Roskilde, DK) for 1 h at room temperature. Next, the cells were washed with PBS thrice and left to dry. Finally, the slides were mounted with Immu‐Mount (Fisher Scientific, Roskilde, DK) and visualized with EVOS® FL Cell Imaging System. Images were captured using a 10× objective lens with a numerical aperture 0.75. Fluorescence imaging utilized an LED light source, with specific excitation/emission filters for green fluorescent protein (GFP, 488 nm excitation/509 nm emission) and DAPI (358 nm excitation/461 nm emission).

### 4.2 Time‐dependent degradomics analysis of MMP9 in wt and COSMC ko MDA‐MB‐231 cells

#### 4.2.1 Secretome preparation from MDA‐MB‐231 cells

MDA‐MB‐231 wt and MDA‐MB‐231 COSMC ko cells were cultured in DMEM media, supplemented with 1% penicillin/streptomycin and 10% FBS, at 37°C in a 5% CO_2_ atmosphere. To collect the cell culture's secretome, 70% confluent cells in T175 flasks were washed three times with 15 mL of PBS and incubated with 15 mL of DMEM media without phenol red (Merck Life Science A/S, Søborg, DK) and serum at 37°C in a 5% CO_2_ atmosphere, as previously described (Schlage et al., 2014). After 24 h, the media were collected and centrifuged for 5 min at 1000*g* to remove cell debris. Next, protease inhibitors (PMSF, Roche, Basel, CH) and 1 mM ethylenediaminetetraacetic acid (EDTA, Merck Life Science A/S, Søborg, DK) were added to the collected media, followed by centrifugation at 5000*g* for 30 min at 4°C to remove smaller debris. The medium was sterile filtered and concentrated by ultrafiltration, followed by buffer exchange to 50 mM of 4‐(2‐hydroxyethyl)‐1‐piperazineethanesulfonic acid (HEPES, Merck Life Science A/S, Søborg, DK) buffer pH 7.8 using Amicon Ultra‐15 3 kDa‐cutoff centrifugal filter units (Merck Life Science A/S, Søborg, DK) to a final volume of about 1 mL. Total protein concentration was determined by Bradford assay company (Bio‐Rad Laboratories, København, DK), and the secretome samples were stored at −80°C before further processing.

#### 4.2.2 16plexTMT‐TAILS workflow

The MDA‐MB‐231 wt and COSMC ko secretomes were incubated with activated recombinant human MMP9 (R&D Systems, Minneapolis, USA) at an enzyme: protein ratio of 1:100 (w:w) in presence of 10 mM calcium chloride (CaCl_2_, Merck Life Science A/S, Søborg, DK) and 100 mM sodium chloride (NaCl, Merck Life Science A/S, Søborg, DK) and sampled at multiple time points (50 μg per time point) up to 16 h at 37°C. Samples for control at 0 and 16 h were incubated with an equivalent buffer volume without MMP9. The digestion at each time point was quenched with 2.5 M guanidine hydrochloride (GuHCl, Merck Life Science A/S, Søborg, DK), 250 mM HEPES pH 7.8. Next, we followed the TMT‐TAILS workflow, as previously described (Madzharova, Sabino, et al., 2019). Briefly, the protein samples from the MMP9‐digested MDA‐MB‐231 secretomes were denatured by incubation for 15 min at 65°C. Next, the cysteine residues of each sample were reduced by adding 3.5 mM tris‐(2‐carboxyethyl)‐phosphine (TCEP, Thermo Fisher Scientific, Roskilde, DK) and incubation for 45 min at 65°C, and then alkylated by adding 5 mM of chloroacetamide (CAA, Merck Life Science A/S, Søborg, DK) for 30 min at 65°C. The proteins in each sample were labelled at a 1:4 protein: TMT (w/w) ratio with TMT reagents (ThermoFisher Scientific, Roskilde, DK) for 1.5 h at room temperature, after which the labelling reactions were quenched with 100 mM ammonium bicarbonate (NH_4_HCO_3_, Merck Life Science A/S, Søborg, DK) for 30 min at room temperature. The labelled samples were then pooled together and precipitated by adding seven sample volumes of ice‐cold acetone (Merck Life Science A/S, Søborg, DK) and one sample volume of ice‐cold methanol (MeOH, Merck Life Science A/S, Søborg, DK) and incubated for 2 h at −80°C. The mixed sample was centrifuged at 5000*g* at 4°C for 30 min, washed with 5 mL ice‐cold MeOH, and centrifuged again. The pellet was air‐dried, resuspended in 100 mM sodium hydroxide (NaOH, Merck Life Science A/S, Søborg, DK), and adjusted with 1 M HEPES, pH 7.8 to 1 mg/mL protein in 100 mM HEPES, pH 7.8. The samples were digested with trypsin (1:100 enzyme: protein ratio (w/w), Promega, Madison, WI) for 16 h at 37°C. Next, 10% of the digested proteomes were removed before N‐terminal enrichment (preTAILS sample) and stored at −20°C. The pH of the remaining sample was adjusted to pH 6–7 and incubated with fourfold excess of a hyperbranched aldehyde‐derivatized polyglycerol (HPG‐ALD) polymer (https://ubc.flintbox.com/technologies/888fc51c-36c0-40dc-a5c9-0f176ba68293) in presence of 50 mM sodium cyanoborohydride (NaCNBH_3_, Sigma‐Aldrich, North Ryde, Australia) for 16 h at 37°C. The unbound peptides were recovered by filtration using Amicon Ultra‐0.5 mL Centrifugal Filter Units (30 kDa cut‐off) at 10,000*g* for 10 min at room temperature, whereas the HPG‐ALD polymer bound to the fully tryptic peptides remained in the filter. The polymer was washed with 30 μL 100 mM NH_4_HCO_3_ and centrifuged again. The flow‐throughs of the enriched N‐terminal peptides were combined (TAILS sample) and stored at −20°C until peptide fractionation.

#### 4.2.3 Peptide fractionation and liquid chromatography–tandem mass spectrometry (LC–MS/MS)

The preTAILS and TAILS samples were fractionated using a Dionex UltiMate 3000 UHPLC (ThermoFisher Scientific) coupled with an Acclaim™ PA2 nano HPLC column (3 μm, 150 × 0.3 mm, Thermo Scientific). Before being loaded onto the column, the samples were resuspended in 5 mM NH_4_HCO_3_, pH 10, after which the fractionation gradient was commenced as follows: 2 min 5% B; 50 min 35% B; 58 min 70% B; 65 min 70% B; 70 min 5% B with eluent A (5 mM NH_4_HCO_3_) and eluent B (100% acetonitrile, ACN, Merck Life Science A/S, Søborg, DK) were used at a flow rate of a 5 μL/min. The peptide fractions were loaded to Evotips according to the manufacturer's instructions. The fractions were analyzed on a Q Exactive HF‐X mass spectrometer (ThermoFisher, Bremen, DE) coupled with an LC Evosep One system (EvoSep, Odense, DK). The samples were analyzed with a pre‐programmed 44 min gradient per injection using Acclaim™ PepMap™ RSLC C18 column (2 μm, 75 μm × 150 mm, ThermoFisher Scientific) at room temperature. Data were recorded in data‐dependent acquisition (DDA) mode. A precursor MS1 scan (m/z 350–2000) was acquired at a resolution of 120,000 with an automatic gain control (AGC) target 3e6 and a maximum fill time of 50 ms. The 20 most abundant precursor ions were selected from each MS scan for a subsequent higher‐energy collision‐induced dissociation (HCD) fragmentation with a normalized collision energy (NCE) of 30%. Fragmentation was performed at resolution 45,000 with an AGC target of 1e5 and an injection time of 96 ms, using a precursor isolation window of 0.7 m/z and a dynamic exclusion of 20 s after a single isolation and fragmentation of a given precursor.

#### 4.2.4 Data analysis

Raw files were searched by Sequest HT from Proteome Discoverer v.2.4 (ThermoFisher Scientific, Waltham, MA) against the human UniProt database (taxid: 9606, release 2017_10). For N termini profiling, the following parameters were used for database searches: semi‐ArgC for enzyme specificity, allowing one missed cleavage; carbamidomethyl (C) and TMTpro (K) as fixed modifications, and acetyl (N‐term), TMTpro (N‐term), pyroQ (N‐term), deamidation (NQ), oxidation (M), were set as variable modifications. Precursor mass error tolerance of 10 ppm and fragment mass error at 0.02 Da. Percolator was used for decoy control and FDR estimation (0.01 high confidence peptides, 0.05 medium confidence). For data normalization, the quantification values for each peptide were normalized to the median value of the entire correspondent channel of the pre‐TAILS dataset. For protein‐level analyses, data recorded from the preTAILS sample were searched using the same parameters as for N termini profiling but with complete ArgC specificity, normalization to total peptide amount but excluding N‐terminally TMTpro modified peptides, and scaling on all averages. Based on the quality inspection, quantitative data from 14 of the 16 TMT reporter channels (128C and 132C channels were excluded) were included in subsequent analyses. Positional annotation of N‐terminal peptides for cleavage sites and known Merops events was performed using Clipper 2.0 (Kalogeropoulos et al., 2023; Rawlings & Bateman, 2021). Functional enrichment analysis at the protein level was done with the help of STRING v11 and proteins with differential abundance between conditions determined using CARMAweb 1.6 web interface (Rainer et al., 2006; Szklarczyk et al., 2019). Volcano plots were generated using Microsoft Excel, and heatmaps were plotted in MeV 4.8 (Saeed et al., 2003). For curve fitting and plotting we used GraphPad Prism 8.4.1 and IceLogo 1.3.8 for the generation of specificity logos (Colaert et al., 2009).

### 4.3 Quantitative proteomics analysis of secretomes from wt and COSMC ko cells using data‐independent acquisition (DIA)

#### 4.3.1 Sample preparation and LC–MS/MS

Protein samples (20 μg per condition) from wt and COSMC ko MDA‐MB‐231 secretomes in 4 M GuHCl, 250 mM HEPES pH 7.8 were reduced with 10 mM TCEP and alkylated with 40 mM CAA for 10 min at 95°C. Next, the samples were digested with trypsin at a 1:20 enzyme‐to‐protein ratio (w/w) for 16 h at 37°C. After quenching the digestion, the samples were prepared for analysis by loading them onto Evotips according to the manufacturer's instructions. The samples were analyzed on an Orbitrap Exploris™ 480 mass spectrometer (ThermoFisher Scientific, Bremen, DE) coupled with an LC Evosep One system, with a pre‐programmed Whisper100 10SPD method using an Acclaim™ PepMap™ RSLC C18 column (1.9 μm, 75 μm × 150 mm, ThermoFisher Scientific). The mass spectrometer operated in positive mode, integrated with a FAIMS Pro interface, where the compensation voltage was adjusted to −45 V. MS scans were acquired at 120,000 resolution and a scan range (m/z) of 400–1000, with an AGC of 300% and the maximum injection time on auto setting. The DIA isolation windows covered a scan range of 400–1000 m/z. Precursor fragmentation was facilitated by HCD at an NCE of 32% at a resolution of 60,000, AGC set to 1000%, and the maximum injection time on auto setting.

#### 4.3.2 Data analysis

Raw data files were processed using Spectronaut v.17 (Biognosys AG, Zurich, CH) using DirectDIA (Deep) mode, querying against the human UniProt database (taxonomy ID: 9606, released October 2017) using modified BGS factory settings. For the database search, the following parameters were used: trypsin for enzyme specificity, allowing two missed cleavages; carbamidomethyl (C) as fixed modification, and acetyl (Protein N‐term) and oxidation (M) were set as variable modifications. The quantification level was set to MS1 using only proteotypic peptides. Missing values were imputed using the background signal. Subsequently, protein and peptide quantification data were extracted, further analyzed, and visualized using GraphPad Prism version 9.5.

### 4.4 Examining CD74 processing by cathepsin S using parallel reaction monitoring (PRM)

#### 4.4.1 Sample preparation and LC–MS/MS

2 μg samples of recombinant human CD74 protein, with distinctions in their expression systems: one glycosylated version from HEK cells (Abcam, ab174039, Cambridge, UK) and a non‐glycosylated variant from *E. coli* (Abcam, ab156328, Cambridge, UK), were incubated with activated recombinant Cathepsin S (CTSS, R&D Systems, Minneapolis, MN) at an enzyme/protein ratio (m/m) of 1:100 for 4 h in 100 mM HEPES, pH 7.8, at 37°C. The reaction was quenched by reduction with 10 mM TCEP and alkylation with 40 mM CAA at 95°C for 10 min. Subsequently, the samples were digested with trypsin at an enzyme/protein ratio (m/m) of 1:20 for 16 h at 37°C. Before PRM analysis, peptide samples were desalted using SOLAμ™ SPE Plates (ThermoFisher Scientific, Roskilde, DK), following the manufacturer's instructions. The samples were analyzed on a Q Exactive mass spectrometer (ThermoFisher Scientific, Bremen, DE) coupled to an EASY‐nLC™ 1200 chromatography system (ThermoFisher Scientific). Peptides were separated on an Acclaim™ PepMap™ RSLC C18 column (2 μm particle size, 75 μm × 150 mm length, ThermoFisher Scientific). The mobile phase consisted of buffer A (0.1% FA) and buffer B (ACN in 0.1% FA). A 70 min gradient was employed as follows: 6%–23% buffer B over 43 min, 23%–38% buffer B over 12 min, 38%–60% buffer B over 5 min, and 60–95% buffer B over 10 min, at a constant flow rate of 250 nL/min. Data were acquired in unscheduled PRM mode with distinct settings for full MS and targeted PRM scans. For full MS scans, the resolution was set at 70,000, with an AGC target of 3e6 and a maximum injection time of 20 ms. Targeted PRM scans were acquired at a resolution of 35,000, with an AGC target of 3e6. Each peptide was isolated using a 1.2 m/z isolation window, with a maximum injection time of 250 ms, and subsequently fragmented by HCD with an NCE of 28%.

#### 4.4.2 Data analysis

The PRM data were analyzed using Skyline version 23.1 (MacCoss Lab, University of Washington, Seattle, WA) (Pino et al., 2020). Target peptides were imported with the filter parameters set to a resolution of 60,000 at 400 m/z for MS1 Orbitrap scans and a resolution of 30,000 at 400 m/z for MS/MS targeted scans. Each precursor mass was configured to monitor at least five transitions. The selection of elution profiles for the most confident peptides was based on a dot product threshold of at least 0.90, with a maximum allowed deviation of 5 ppm for the transitions. Peaks that met these criteria were matched against the in‐house target library for further analysis.

### 4.5 Glycoproteomics analysis

#### 4.5.1 Sample preparation and LC–MS/MS

A total of 5 μg of human recombinant CD74 protein, expressed in HEK cells, was reduced with 10 mM TCEP and alkylated with 40 mM CAA for 10 min at 95°C. Next, CD74 was digested with trypsin (1:20, w/w) overnight at 37°C. The resulting CD74 peptide mixture was desalted using SOLAμ™ SPE Plates, following the manufacturer's instructions. Next, the sample was analyzed on an Orbitrap Fusion Tribrid Mass Spectrometer (Thermo Fisher Scientific, Bremen, DE) coupled to an EASY‐nLC™ 1200 chromatography system (ThermoFisher Scientific). Peptides were separated on an Acclaim™ PepMap™ RSLC C18 column (2 μm particle size, 75 μm × 150 mm length, ThermoFisher Scientific). The mobile phase consisted of buffer A (0.1% FA) and buffer B (ACN in 0.1% FA). A 70‐min gradient was employed as follows: 6%–23% buffer B over 43 min, 23%–38% buffer B over 12 min, 38%–60% buffer B over 5 min, and 60%–95% buffer B over 10 min, at a constant flow rate of 250 nL/min. Data acquisition was performed in DDA mode with the following configurations: Full MS scans were conducted in the Orbitrap at a resolution of 120,000 at m/z 200, and the AGC target was set to 4e5 ions, with a maximum injection time of 50 ms. The most intense precursor ions underwent MS/MS fragmentation via HCD with an NCE of 30%. MS/MS scans were acquired at a resolution of 30,000 at m/z 200, with an AGC target of 2e5 ions and a maximum injection time of 120 ms. The acquisition strategy incorporated a product ion‐triggered re‐isolation of the precursor ion if HexNAc oxonium ions (138.0545, 204.0867, and 366.1396 m/z) were identified among the top 20 fragment ions in the HCD‐MS/MS spectrum. These re‐isolated precursor ions were then analyzed using electron transfer dissociation (ETD) and collision‐induced dissociation (CID) MS/MS. The ETD‐MS/MS analysis parameters were set to a resolution of 30,000, an AGC of 2e5 ions, an injection time of 120 ms, and a quadrupole isolation width of 2 m/z. CID MS/MS scans were performed in the ion trap with a ‘Rapid’ scan rate, an AGC target of 2e4 ions, an injection time of 70 ms, an NCE of 30%, and a quadrupole isolation width of 2 m/z.

#### 4.5.2 Data analysis

The raw data were analyzed using Byonic v2.6.46 (Protein Metrics, USA) against a combined protein and glycan database (Bern et al., 2012). The following parameters were used for database search: canonical human CD74 sequence (UniProtKB, P04233), trypsin for enzyme specificity, allowing up to two missed cleavages; carbamidomethyl (C) was set as a fixed modification, whereas oxidation (M), deamidation (N), and *O*‐glycosylation of Thr/Ser residues, employing a predefined *O*‐glycan database, comprised of 78 common mammalian *O*‐glycans, were set as variable modifications. All searches were filtered to <1% false discovery rate (FDR) at the protein level.

## AUTHOR CONTRIBUTIONS

**Elizabeta Madzharova:** Investigation; writing – original draft; methodology; validation; writing – review and editing; formal analysis; visualization; data curation. **Fabio Sabino:** Supervision; formal analysis; investigation. **Konstantinos Kalogeropoulos:** Writing – review and editing. **Chiara Francavilla:** Writing – review and editing. **Ulrich auf dem Keller:** Conceptualization; funding acquisition; supervision; formal analysis; writing – review and editing; resources; project administration.

## CONFLICT OF INTEREST STATEMENT

All authors declare no conflict of interest.

## Supporting information


**Table S1:** Proteins identified in preTAILS sample.

**Table S2:** N‐termini identified in preTAILS sample.

**Table S3:** Proteins identified in TAILS sample.

**Table S4:** N‐termini identified in TAILS sample.

**Table S5:** neo‐N‐termini identified in TAILS sample.

**Table S6:** Proteins identified and quantified with internal tryptic peptides in preTAILS.

**Table S7:** List of functional enrichment analysis of down‐regulated proteins in COSMC ko, using STRING v11 and Reactome pathways.

**Table S8:** Proteins identified and quantified by DIA.

**Table S9:** Quantification of VTSQNLQLENLR peptide, derived from CD74 by PRM.

**Table S10:** Glycopeptides identified from human recombinant CD74, expressed in HEK cells.

**Figure S1:** Sanger sequencing results for wt and COSMC ko MDA‐MB‐231 cells.
**Figure S2.** Principal Component Analysis (PCA) of wt and COSMC ko MDA‐MB‐231 proteomics data.
**Figure S3.** Pearson correlation analysis of wt and COSMC ko MDA‐MB‐231 proteomics data.
**Figure S4.** Validation of CTSS‐mediated cleavage of CD74 via targeted degradomics.
**Figure S5.** MMP9 protein abundance across different samples.

## Data Availability

The mass spectrometry data have been deposited to the ProteomeXchange Consortion (Deutsch et al., 2017) via the PRIDE (Perez‐Riverol et al., 2019) partner repository with the following accession codes: PXD050580 (16plexTMT‐TAILS dataset), PXD050581 (DIA dataset), PXD050644 (PRM dataset), and PXD050647(Glycoproteomics dataset).
