# Supplementary material for: Substrate O‐glycosylation actively regulates extracellular proteolysis
Source: Protein Sci. 2024 Jul 29;33(8):e5128. doi: 10.1002/pro.5128 (PMC11285871; doi:10.1002/pro.5128)
Supplement: Supplementary file 11 — Figure S1: Sanger sequencing results for wt and COSMC ko MDA‐MB‐231 cells. Figure S2. Principal Component Analysis (PCA) of wt and COSMC ko MDA‐MB‐231 proteomics data. Figure S3. Pearson correlation analysis of wt and COSMC ko MDA‐MB‐231 proteomics data. Figure S4. Validation of CTSS‐mediated cleavage of CD74 via targeted degradomics. Figure S5. MMP9 protein abundance across different samples. [file PRO-33-e5128-s004.docx]

**Supplementary Material**

**Substrate O-glycosylation actively regulates extracellular proteolysis**

Elizabeta Madzharova^1,*^, Fabio Sabino^1^, Konstantinos Kalogeropoulos^1^, Chiara Francavilla^1,*^ Ulrich auf dem Keller^1,2^

^1^Department of Biotechnology and Biomedicine, Technical University of Denmark, DK-2800 Kongens Lyngby, Denmark

^2^deceased

**Supplementary Figures and legends**


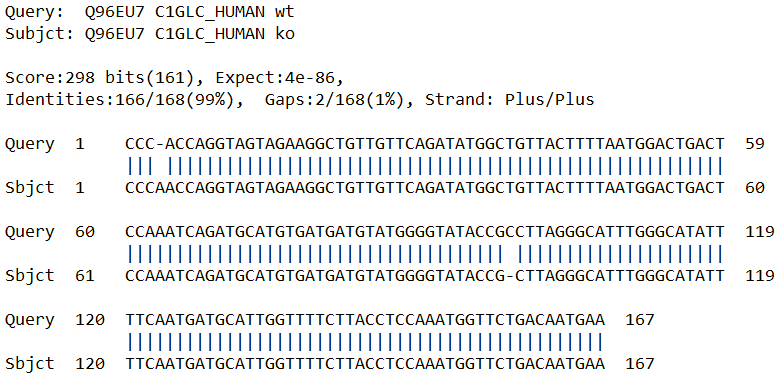


**Supplementary Figure S1. Sanger sequencing results for wt and COSMC ko MDA-MB-231 cells.** Comparative alignment between the wt and COSMC ko sequences reveals a deletion of the nucleotide at position 98. This mutation differentiates the COSMC ko cells from their wt counterparts, providing insight into the genetic alterations induced in COSMC gene by CRISPR Cas9.


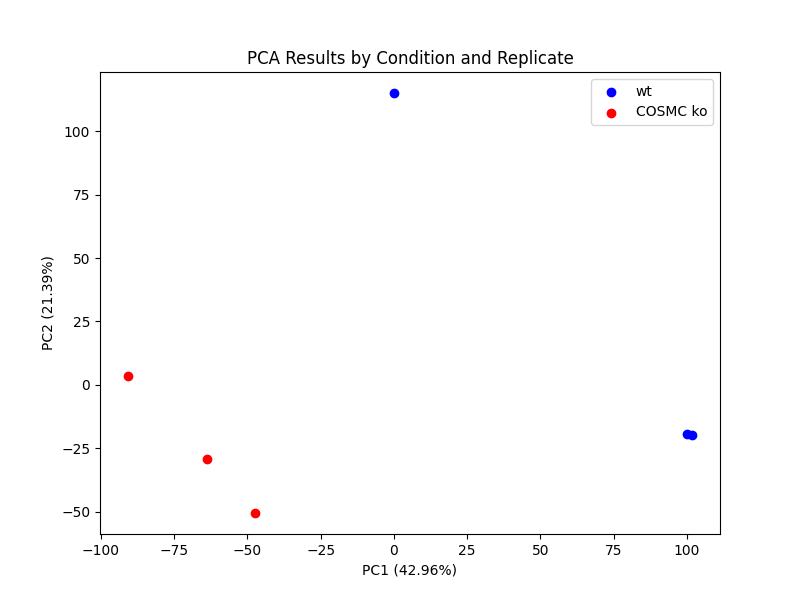
**Supplementary Figure S2. Principal Component Analysis (PCA) of wt and COSMC ko MDA-MB-231 proteomics data**. The scatter plot shows the variance in proteomic profiles between wt and COSMC ko cells, each represented by three technical replicates. The PCA was conducted on log2-transformed, standardized protein expression levels. PC1 and PC2 are plotted on the x and y axes, respectively, with each axis labeled with the percentage of the total variance explained by that component.

**
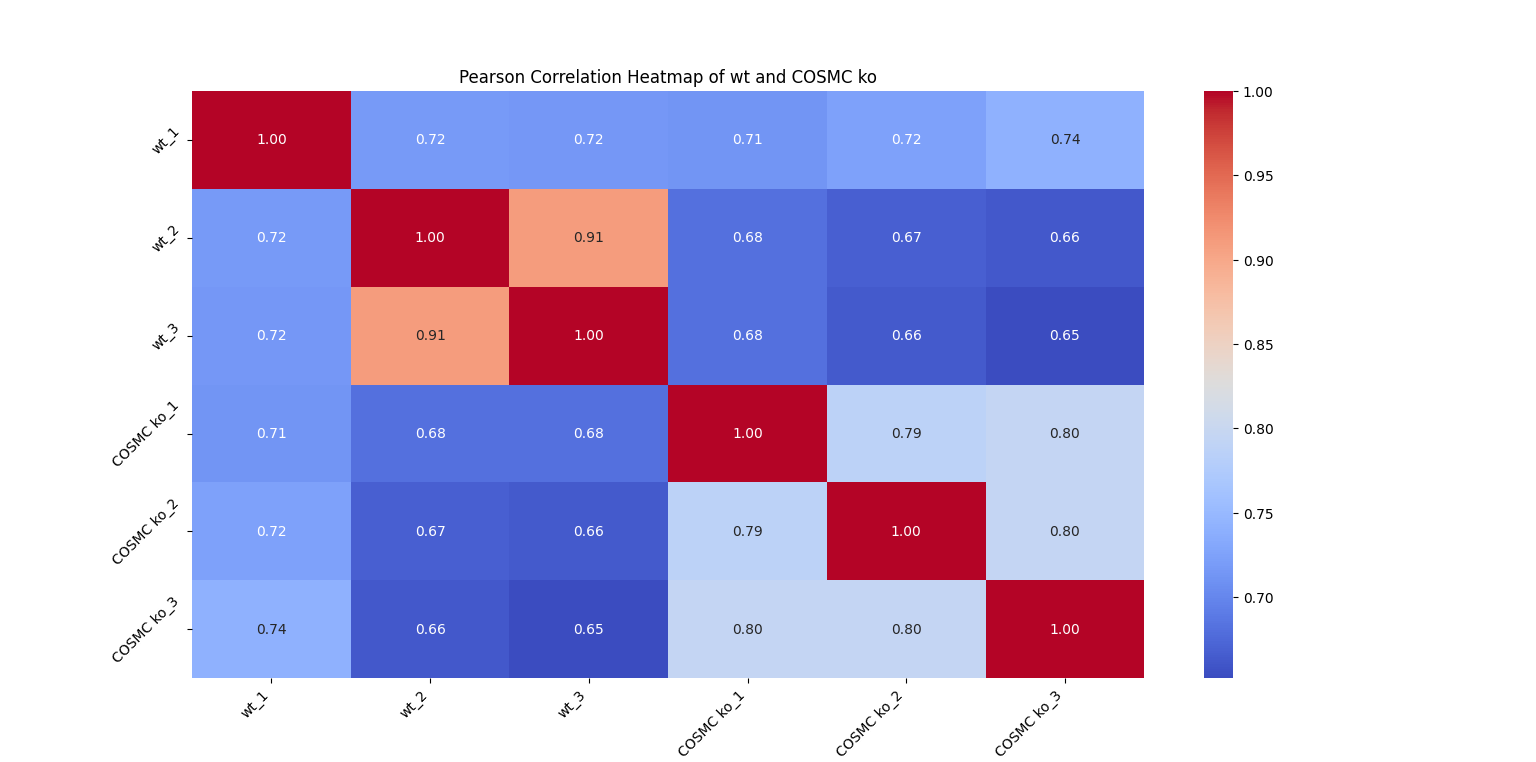
**

**Supplementary Figure S3. Pearson correlation analysis of wt and COSMC ko MDA-MB-231 proteomics data.** The heatmap illustrates Pearson correlation coefficients derived from log2-transformed protein expression levels, where each cell reflects the correlation between pairs of proteomic profiles from wt and COSMC ko cells. These profiles are represented by three technical replicates for each condition, spanning all identified proteins.

**
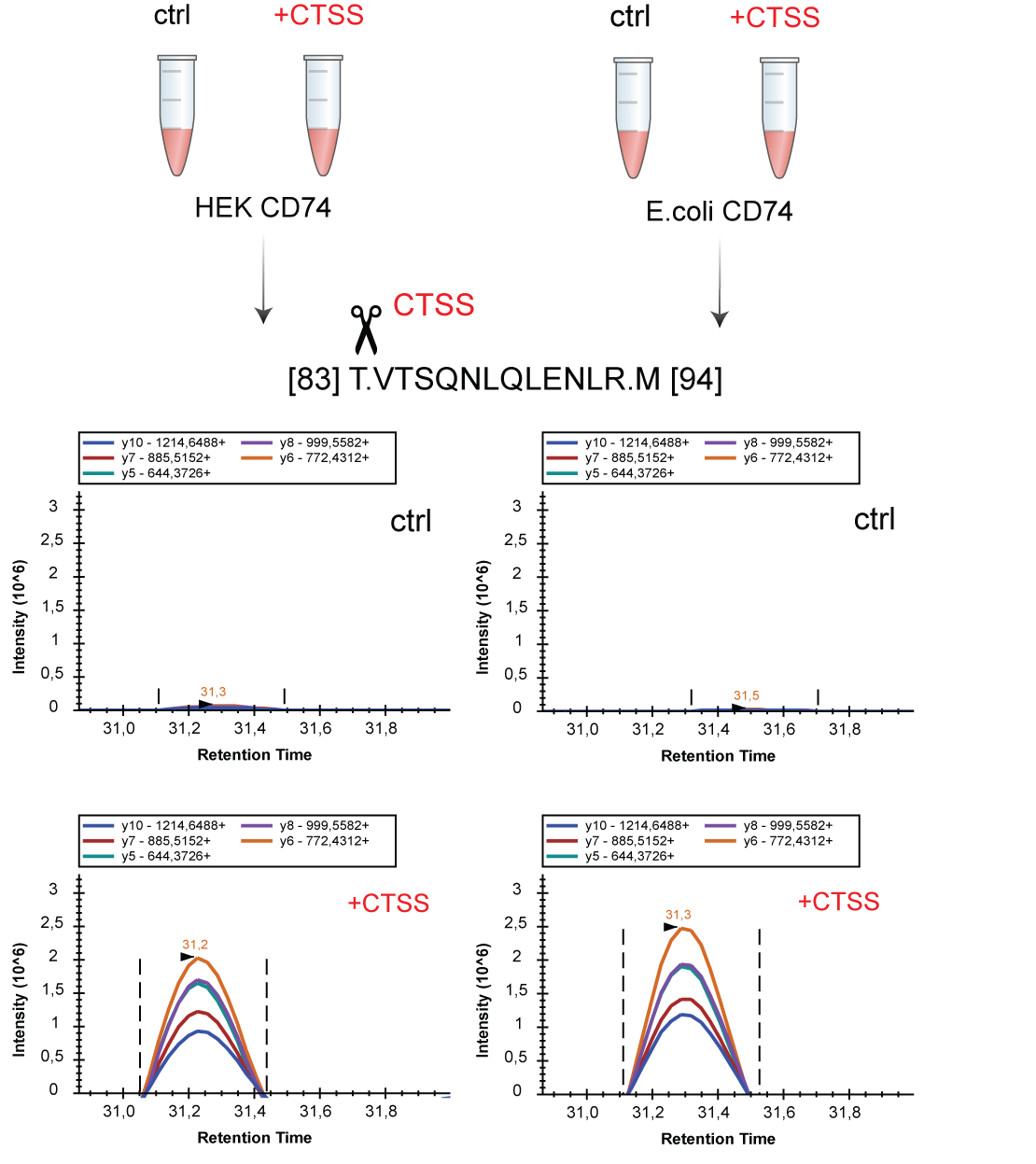
**

**Supplementary Figure S4. Validation of CTSS-mediated cleavage of CD74 via targeted degradomics**. Glycosylated and non-glycosylated recombinant human CD74 proteins from HEK cells and E. coli, respectively, treated with activated CTSS for 4 hours at 37°C. Control samples (ctrl) were untreated. Cleavage assessed by monitoring the semi-tryptic peptide VTSQNLQLENLR using PRM.


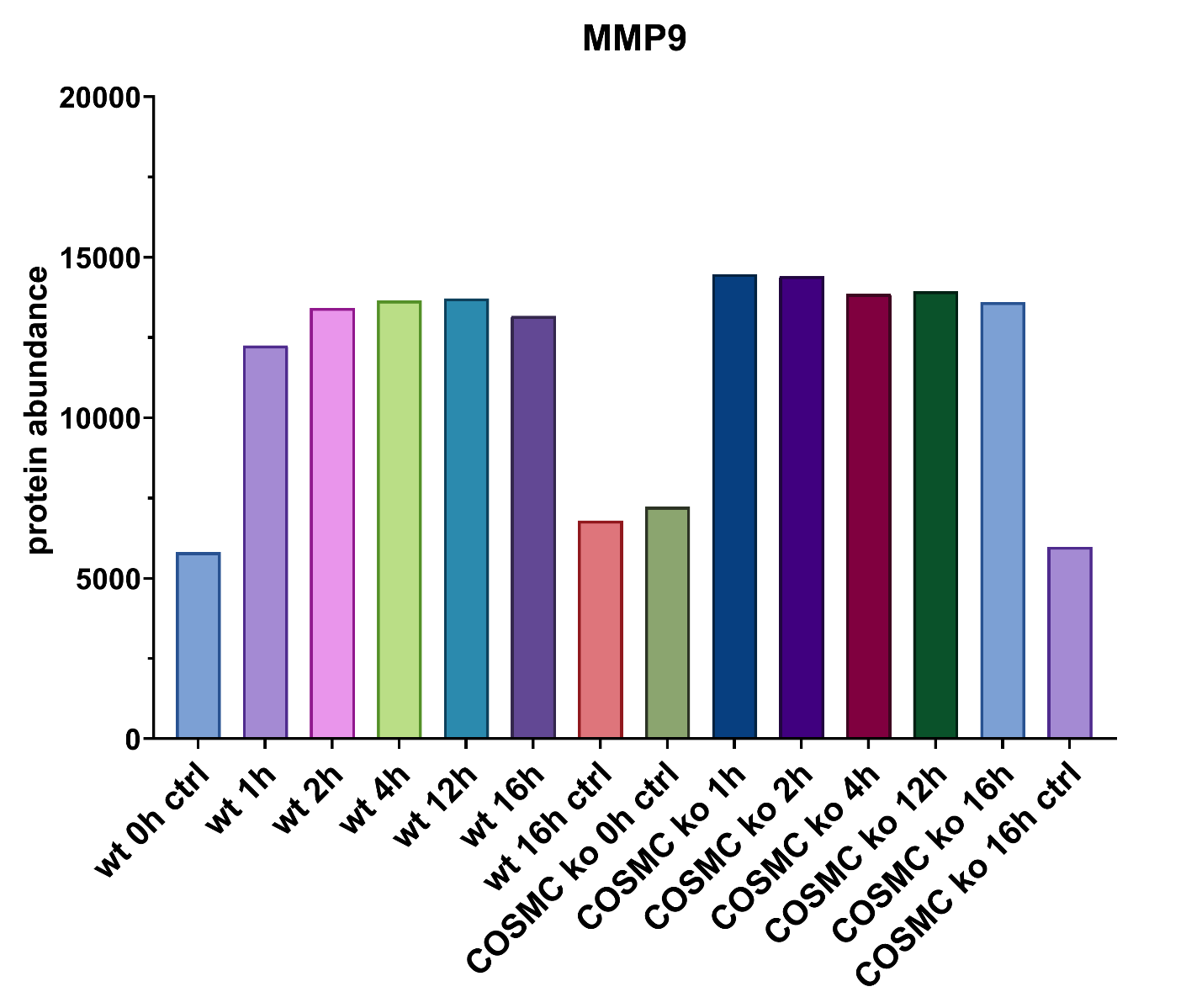


**Supplementary Figure S5. MMP9 protein abundance across different samples**. The bar graph illustrates the protein abundance of MMP9 in wt and COSMC ko samples over a time-series. The data highlights the detectable spike of recombinant MMP9 in the samples, allowing for a comparison of MMP9 levels between the control and experimental samples over time.

**Supplementary Tables**

**Supplementary Table S1**: Proteins identified in preTAILS sample

**Supplementary Table S2:** N-termini identified in preTAILS sample

**Supplementary Table S3:** Proteins identified in TAILS sample

**Supplementary Table S4**: N-termini identified in TAILS sample

**Supplementary Table S5:** neo-N-termini identified in TAILS sample

**Supplementary Table S6:** Proteins identified and quantified with internal tryptic peptides in preTAILS

**Supplementary Table S7:** List of functional enrichment analysis of down-regulated proteins in COSMC ko, using STRING v11 and Reactome pathways

**Supplementary Table S8:** Proteins identified and quantified by DIA

**Supplementary Table S9:** Quantification of VTSQNLQLENLR peptide, derived from CD74 by PRM

**Supplementary Table S10:** Glycopeptides identified from human recombinant CD74, expressed in HEK cells
